# Spontaneous Bacterial Peritonitis in a Patient With Nonportal Hypertensive Ascites

**DOI:** 10.31486/toj.21.0050

**Published:** 2022

**Authors:** Marc Manzo, Parth Desai

**Affiliations:** Department of Hospital Medicine, Ochsner Clinic Foundation, Ochsner Medical Center – West Bank Campus, Gretna, LA

**Keywords:** *Amyloidosis*, *ascites*, *cardiomyopathies*, *immunosuppression therapy*, *kidney failure—chronic*, *peritonitis*

## Abstract

**Background:** Spontaneous bacterial peritonitis (SBP) is a life-threatening condition classically found as a complication of cirrhotic ascites, but it has rarely been documented in a case of nonportal hypertensive ascites.

**Case Report:** We report the case of a 54-year-old male with SBP arising from nonportal hypertensive ascites in the setting of end-stage renal disease and restrictive cardiomyopathy, both secondary to primary amyloidosis (AL type, kappa light chain). Peritoneal fluid analysis showed a serum-ascites albumin gradient of 1.1 g/dL and total fluid protein of 3.6 g/dL consistent with nonportal hypertensive etiology. The patient was managed empirically with intravenous ceftriaxone and intravenous albumin. Additional workup was nondiagnostic for other causes of ascites, and the patient was discharged after a 7-day hospital course.

**Conclusion:** Patients presenting with refractory ascites in the setting of end-stage renal disease, cardiomyopathy, and long-standing immunosuppressive therapy may be at increased risk for SBP despite a high ascitic fluid protein.

## INTRODUCTION

Ascites is the abnormal accumulation of fluid within the peritoneal cavity and is most commonly caused by cirrhosis. Other etiologies include congestive heart failure, renal disease, malignancy, and infectious diseases.^1,2^ Ascites is characterized by the peritoneal fluid serum-ascites albumin gradient (SAAG) and total fluid protein level (Table 1). A SAAG in cirrhosis is >1.1 g/dL, indicating a portal hypertensive etiology. Total fluid protein in cirrhosis is <2.5 g/dL. Conversely, in cardiac and nephrogenic causes of ascites, total fluid protein is >2.5 g/dL.^1,2^

**Table 1.** Peritoneal Fluid Features of Ascites

Ascites Type	Serum-Ascites Albumin Gradient, g/dL	Total Fluid Protein, g/dL
Cirrhotic	>1.1	<2.5
Cardiac	>1.1	>2.5
Nephrogenic	<1.1	>2.5

Cirrhotic ascites is a consequence of portal hypertension. Portal hypertension creates a hyperdynamic circulatory response that causes a reduction in systemic vascular resistance, primarily in the splanchnic arterial circulation.^1,2^ Antidiuretic hormone and the renin-angiotensin-aldosterone system inappropriately retain water and salt. The excess volume transudates through the splanchnic capillaries and hepatic sinusoids and subsequently accumulates in the peritoneum.^1,2^

Noncirrotic causes of ascites are much more uncommon; etiologies include congestive heart failure and end-stage renal disease.^3,4^ The cause of cardiac ascites is congestive hepatopathy. Because the liver receives up to 25% of cardiac output, any cause of right-sided heart failure can result in elevated central and hepatic venous pressures.^3^ Elevated pressures cause impaired hepatic venous outflow and congestion, which results in transudation from the hepatic and portal veins.^3^ Nephrogenic ascites, also known as hemodialysis-associated ascites, is defined as refractory ascites associated with end-stage renal disease without an alternate etiology. Most patients with nephrogenic ascites are on maintenance hemodialysis, but peritoneal dialysis has been recorded as well.^4,5^ The pathogenesis of nephrogenic ascites is unclear and likely multifactorial. Possible causes are elevated hepatic venous pressures, volume overload, increased peritoneal membrane permeability secondary to uremic toxins, and impaired lymphatic drainage.^5,6^

The general treatment for ascites is centered around strict volume control with fluid/salt restriction, diuresis, large-volume paracentesis, and discontinuation of medications that reduce renal perfusion, such as nonsteroidal anti-inflammatory drugs, angiotensin-converting enzyme inhibitors, angiotensin receptor blockers, and beta blockers.^6,7^ The only definitive long-term treatment for nephrogenic ascites is renal transplantation; however, dialysis is a short-term solution.^6,7^

One life-threatening complication of ascites is spontaneous bacterial peritonitis (SBP), an infection of ascitic fluid without a secondary intra-abdominal source. The pathophysiology of SBP is the translocation of gut bacteria into ascitic fluid, with 75% of SBP arising in patients with cirrhotic ascites.^8^ The diagnosis is made by an ascitic fluid neutrophil count >250/mm^3^.^7^ Management begins with empiric antibiotics—third-generation cephalosporins—that are tailored according to ascitic culture sensitivities. Cefotaxime, ceftriaxone, and ceftazidime have been shown to cover approximately 95% of ascitic and gut flora, with the most common pathogens being *Escherichia coli, Klebsiella pneumoniae*, and *Staphylococcus aureus*.^7^ In patients with renal dysfunction, ceftriaxone and albumin should be the empiric treatment of choice. Of note, culture-negative ascitic fluid occurs approximately 60% of the time and may present in a clinically similar way to a culture-positive SBP presentation.^8^ Culture-negative ascitic fluid should be treated empirically with antibiotics, as culture-positive and culture-negative patients have similar mortality rates.^8^ Providers must maintain a high level of clinical suspicion for SBP even in cases of nonportal hypertensive ascites, as mortality can increase from 10% to 50% if empiric antibiotic treatment is delayed.^8^

## CASE REPORT

A 54-year-old male presented to the emergency department (ED) with abdominal distention and diffuse abdominal pain associated with dyspnea, chills, and orthopnea. The patient had a medical history of primary amyloidosis (AL type, kappa light chain), restrictive cardiomyopathy, end-stage renal disease (on long-term hemodialysis), and refractory ascites. The patient's cardiomyopathy and end-stage renal disease were both complications of his primary amyloidosis. He was previously treated with 4 cycles of CyBorD (cyclophosphamide, bortezomib, dexamethasone) and then transitioned to maintenance bortezomib every 2 weeks.

On arrival at the ED, the patient was afebrile, but vital signs were remarkable for blood pressure 89/50 mmHg, heart rate 108/min, and respiratory distress (respiratory rate 25/min, oxygen saturation 93%). Physical examination revealed a tense distended abdomen without guarding and mild pitting edema of bilateral lower extremities.

Laboratory workup (Table 2) was significant for leukocytosis. Liver chemistry was unremarkable. Brain natriuretic peptide level of >4,900 pg/mL, which is chronically elevated in cardiac amyloidosis, and troponin level ruled out infarction. Electrocardiogram and chest x-ray were negative for acute cardiac or pulmonary processes.

**Table 2.** Laboratory Results on Admission

Test	Result	Reference Range
White blood cells, K/μL	23.34	3.9-12.7
Brain natriuretic peptide, pg/mL	>4,900	0-99
Lactate dehydrogenase, U/L	577	110-260
Troponin I, ng/mL	1.4	0.00-0.02
Aspartate aminotransferase, U/L	28	10-40
Alanine transaminase, U/L	16	10-44
Total bilirubin, mg/dL	0.7	0.1-1.0

Urgent ultrasound-guided paracentesis drained 7.35 L of amber fluid. Fluid findings revealed polymorphonuclear leukocyte (PMN) count of 3,899 cells/mm^3^, total fluid protein of 3.6 g/dL, and a SAAG of 1 g/dL, establishing a diagnosis of SBP. The patient was treated empirically with intravenous (IV) ceftriaxone 2 g daily and IV albumin. On day 4 of hospitalization, a second paracentesis was performed because of continued abdominal distension and to assess treatment response. Paracentesis extracted 1.4 L of peritoneal fluid; the analysis reported a PMN count of 2,004 cells/mm^3^, total fluid protein of 3.6 g/dL, and a SAAG of 1.1 g/dL. Ascitic fluid cultures were negative for bacterial growth, so ceftriaxone was continued. Blood cultures were also negative.

Abdominal computed tomography ruled out secondary causes of peritonitis and was negative for perforation, bowel wall thickening, and suspicious peritoneal lesions. Ultrasound of the liver was negative for cirrhotic features or portal hypertension and demonstrated adequate flow through the hepatic arteries. Echocardiography showed grade III diastolic dysfunction, ejection fraction of 70%, and infiltrative disease consistent with primary amyloidosis. Estimated pulmonary artery systolic pressure was mildly elevated at 19 mmHg, and central venous pressure was 8 mmHg.

After a 7-day hospital course, the patient improved clinically with resolution of his abdominal pain and ascites after paracentesis, IV ceftriaxone, IV albumin, and maintenance hemodialysis. The patient was discharged with prophylactic oral ciprofloxacin 500 mg every 24 hours for 7 days.

When the patient followed up with his primary care provider 2 weeks after discharge, his ascites had resolved. Because of concerns for increasing light chains, the patient was recommended to restart his CyBorD regimen and add daratumumab.

## DISCUSSION

For our patient, a definitive diagnosis of nephrogenic ascites or cardiac ascites could not be made. With a total fluid protein level >2.5 g/dL and borderline SAAG of 1 g/dL and 1.1 g/dL, we suspect the pathogenesis was multifactorial. The incidence of nephrogenic ascites has been estimated to be 0.7% to 20% in patients with end-stage renal disease,^4^ while cardiac ascites has an incidence of 5%.^9^ Nephrogenic ascites is a diagnosis of exclusion, and portal hypertensive, infectious, and malignant processes must be ruled out. Furthermore, in the setting of cardiac dysfunction, a diagnosis of cardiac ascites cannot be excluded.^5^

Interestingly, our patient had a total fluid protein level of 3.6 g/dL, which is protective against SBP. Total fluid protein levels >2.5 g/dL are opsonic and generate a robust innate immune response, making an episode of SBP less likely for patients with both cardiac and nephrogenic ascites.^10^ General risk factors for a first-time episode of SBP are low ascitic protein (<1 g/dL), elevated serum bilirubin, and advanced stages of cirrhosis.^11^

We speculate that the protective effects of higher protein content were negated by our patient's long-term immunosuppressive therapy. Horn et al reported a similar case, a patient with acute renal transplant rejection that led to ascites complicated by SBP.^12^ The patient had total fluid protein of 5.1 g/dL. The patient's immunosuppressive regimen consisted of cyclosporin A and prednisone, and the patient's cyclosporine trough at the time of presentation was therapeutic at 100 ng/mL. Horina et al reported a case involving a patient who received chronic hemodialysis for diffuse glomerulonephritis (World Health Organization class IV) secondary to systemic lupus erythematosus.^13^ The patient had total ascitic fluid protein of 5.2 g/dL. She was reportedly on immunosuppressive therapy prior to beginning renal replacement therapy, but her medication regimen was not described. Similarly, our patient had previously received 4 cycles of CyBorD and was on maintenance bortezomib.

SBP has also been reported in association with cardiac ascites. Canakis et al reported the case of an 85-year-old male with cardiac ascites complicated by SBP with a peritoneal fluid analysis demonstrating a SAAG of 1.9 g/dL and total fluid protein of 3.6 g/dL.^9^ The patient had systolic dysfunction with an ejection fraction of 35% to 40% with global hypokinesis. The patient was elderly with multiple comorbidities, but no immunosuppressive medication was reported. The Canakis et al report has some resemblance to our case with the primary difference being that our patient had a preserved ejection fraction with severe diastolic dysfunction. These cases are rare but have important consequences because of the high mortality of SBP if the diagnosis is delayed.^8^

Another risk factor to consider for SBP in patients with cardiac ascites is the gut hypothesis, which states that patients with congestive heart failure and reduced cardiac output develop chronic congestion and intestinal ischemia-reperfusion damage, subsequently leading to intestinal hypoxia, hypercapnia, and local pH changes, all of which are virulence activators for local gastrointestinal microorganisms.^14^ This type of chronic intestinal damage leads to translocation of the gut microbiome into the ascitic fluid.^14^ Although our patient had substantial cardiac diastolic dysfunction, we favor immunosuppression and nephrogenic ascites as the patient's major risk factors for SBP. On echocardiography, his central venous pressure was within normal limits at 8 mmHg, ejection fraction was preserved at 70%, and pulmonary artery systolic pressure was only mildly elevated at 19 mmHg. These findings led us to think that congestion was minimal.

One limitation to our case study is that we did not have the full history of our patient's CyBorD regimen. Also, the reporting on cardiac ascites and nephrogenic ascites is limited, and nomenclature is inconsistent. In retrospect, after further review of cardiac ascites, we should have kept in mind the possibility of spontaneous fungal peritonitis (SFP). SFP is less common than SBP but has a higher mortality rate because of late recognition.^15,16^ SFP occurs primarily in patients who have a history of liver cirrhosis. The reported prevalence is 10% in patients who are critically ill with liver cirrhosis.^15^ Our patient responded clinically to ceftriaxone, so suspicions were low for SFP.

## CONCLUSION

Patients presenting with refractory ascites in the setting of end-stage renal disease, cardiomyopathy, and long-standing immunosuppressive therapy may be at increased risk for SBP despite a high ascitic fluid protein. The pathogenesis is uncertain and considerably rare due to the high protein levels that are protective of the ascitic fluid.

## Notes

This article meets the Accreditation Council for Graduate Medical Education and the American Board of Medical Specialties Maintenance of Certification competencies for Patient Care and Medical Knowledge.

## References

[1] Bloom S, Kemp W, Lubel J. Portal hypertension: pathophysiology, diagnosis and management. Intern Med J. 2015;45(1):16-26. doi: 10.1111/imj.12590 25230084

[2] Pedersen JS, Bendtsen F, Møller S. Management of cirrhotic ascites. Ther Adv Chronic Dis. 2015;6(3):124-137. doi: 10.1177/2040622315580069 25954497 PMC4416972

[3] Fortea JI, Puente Á, Cuadrado A, Congestive hepatopathy. Int J Mol Sci. 2020;21(24):9420. doi: 10.3390/ijms21249420 PMC7764741 33321947

[4] Hammond TC, Takiyyuddin MA. Nephrogenic ascites: a poorly understood syndrome. J Am Soc Nephrol. 1994;5(5):1173-1177. doi: 10.1681/ASN.V551173 7873726

[5] Han SH, Reynolds TB, Fong TL. Nephrogenic ascites. Analysis of 16 cases and review of the literature. Medicine (Baltimore). 1998;77(4):233-245. doi: 10.1097/00005792-199807000-00002 9715728

[6] Gunal AI, Karaca I, Celiker H, Ilkay E, Duman S. Strict volume control in the treatment of nephrogenic ascites. Nephrol Dial Transplant. 2002;17(7):1248-1251. doi: 10.1093/ndt/17.7.1248 12105248

[7] Biggins SW, Angeli P, Garcia-Tsao G, Diagnosis, evaluation, and management of ascites, spontaneous bacterial peritonitis and hepatorenal syndrome: 2021 practice guidance by the American Association for the Study of Liver Diseases. Hepatology. 2021;74(2):1014-1048. doi: 10.1002/hep.31884 33942342

[8] European Association for the Study of the Liver. EASL clinical practice guidelines on the management of ascites, spontaneous bacterial peritonitis, and hepatorenal syndrome in cirrhosis. J Hepatol. 2010;53(3):397-417. doi: 10.1016/j.jhep.2010.05.004 20633946

[9] Canakis A, Canakis J, Lohani M, Ostrander T. Spontaneous bacterial peritonitis in cardiac ascites: a rare but deadly occurrence. Am J Case Rep. 2019;20:1446-1448. doi: 10.12659/AJCR.915944 31570687 PMC6788488

[10] Runyon BA, Morrissey RL, Hoefs JC, Wyle FA. Opsonic activity of human ascitic fluid: a potentially important protective mechanism against spontaneous bacterial peritonitis. Hepatology. 1985;5(4):634-637. doi: 10.1002/hep.1840050419 4018735

[11] Schwabl P, Bucsics T, Soucek K, Risk factors for development of spontaneous bacterial peritonitis and subsequent mortality in cirrhotic patients with ascites. Liver Int. 2015;35(9):2121-2128. doi: 10.1111/liv.12795 25644943

[12] Horn S, Holzer H, Horina JH. Spontaneous bacterial peritonitis in a patient with nephrogenic ascites during an episode of acute renal transplant rejection. Am J Kidney Dis. 1996;27(3):441-443. doi: 10.1016/s0272-6386(96)90371-6 8604717

[13] Horina JH, Hammer HF, Reisinger EC, Enzinger GF, Holzer H, Krejs GJ. Spontaneous bacterial peritonitis in a hemodialysis patient with systemic lupus erythematosus. Nephron. 1993;65(4):633-635. doi: 10.1159/000187577 8302423

[14] Nagatomo Y, Tang WH. Intersections between microbiome and heart failure: revisiting the gut hypothesis. J Card Fail. 2015;21(12):973-980. doi: 10.1016/j.cardfail.2015.09.017 26435097 PMC4666782

[15] Wang Y, Gandhi S, Attar BM. Spontaneous fungal peritonitis in ascites of cardiac origin. ACG Case Rep J. 2017;4:e42. doi: 10.14309/crj.2017.42 28386572 PMC5373850

[16] Patel D, Iqbal AM, Mubarik A, Spontaneous fungal peritonitis as a rare complication of ascites secondary to cardiac cirrhosis: a case report. Am J Case Rep. 2019;20:1526-1529. doi: 10.12659/AJCR.917757 31619662 PMC6818645

